# Insight into the biological activities of *Fagonia Arabica* L. and its phytochemical constituents

**DOI:** 10.1186/s13568-025-01918-1

**Published:** 2025-08-01

**Authors:** Nashaat N. Mahmoud, Alsayed E. Mekky, Esawy Mahmoud, Mayada M. El-Azab, Muhammad I. Haggag

**Affiliations:** 1 Department of Botany and Microbiology, Faculty of Science, Al-Azhar University, Cairo, 11884 Egypt; 2 Department of Soil and Water Science, Faculty of Agriculture, Tanta University, Tanta , Egypt; 3 Department of Medicinal and Aromatic Plants, Desert Research Center, Cairo, Egypt

**Keywords:** *Fagonia Arabica* L., Phytochemical analysis, Antibacterial, Antioxidant, Anticancer

## Abstract

In Egypt, the genus *Fagonia* is found in all the deserts of Egypt and the Sinai Peninsula. It is widely used to treat many diseases as a home remedy without sufficient scientific studies, especially *Fagonia arabica* L. Therefore, the purpose of this work was to evaluate the comprehensive phytochemical profile and biological evaluation of *F. Arabica* using HPLC method, antimicrobial activity, antioxidants, and cytotoxicity assays. The results showed that the phytochemical components detected in the aerial flowering parts of *F. arabica* were tannins, steroids, terpenoids, diterpenes, glycosides, saponins, flavonoids, phenols, alkaloids, fixed oils, carbohydrates, proteins, and amino acids. HPLC highlighted the presence of fifteen phenolic compounds present in *F. arabica* aqueous extract, and the highest of them were chlorogenic acid, gallic acid, and catechin. The antibacterial study of *F. arabica* extract showed significant inhibitory actions against *Staphylococcus epidermidis*, *Staphylococcus aureus*,* Enterococcus faecalis*,* Escherichia coli*,* Klebsiella pneumoniae*, and *Acinetobacter baumannii* with zone diameters of 20.0, 22.0, 23.0, 21.0, 17.0, and 18.0 mm, respectively. The extract of aerial flowering parts of *F. arabica* showed high value (IC_50_ = 46.25 µg mL^− 1^) in its free radical scavenging activity. The *F. arabica* extract showed low cytotoxicity against the Vero and A549 cell lines with IC_50_ values of 317.09 µg mL^− 1^ and 178.08 µg/mL, respectively. Therefore, the *F. arabica* extract is used as antibacterial and anticancer agents due to their high content of phytochemicals and antioxidants and can be applied in innovative pharmaceutical and medical fields.

**Supplementary Information:**

The online version contains supplementary material available at 10.1186/s13568-025-01918-1.

## Key points

Structure of phenolic compounds identified in *Fagonia arabica* L extract using HPLC.

*Fagonia arabica* L extract contains high levels of phytochemicals and antioxidants.

Using the *Fagonia arabica* L extract as antibacterial and anticancer agents.

**Supplementary Information:**

The online version contains supplementary material available at 10.1186/s13568-025-01918-1.

## Introduction

Natural plant products are closely related to ethnomedical practices with more or less scientific validation in the treatment of many biological diseases particularly in developed nations (Sharifi-Rad et al. 2022a). These herbal remedies are said to be safe and have few to no negative effects, making them a possible alternative to contemporary synthetic medications (Ansari et al. 2023). Many medicinal plant extracts can fight infectious diseases due to their major active components or secondary metabolites (Elkady et al. 2024). Especially phenolic compounds act as antioxidants by donating hydrogen atoms to neutralize free radicals, thus reducing oxidative damage and contributing to cell protection (Kesharwani et al. 2012; Wintola and Afolayan 2015). Phenolic compounds have an anti-hepatotoxic effect as well as an anti-inflammatory effect (El-Azab 2012). These compounds have also been shown to activate the endogenous antioxidant system and inhibition of lipid peroxidation in human red blood cells (Casagrande et al. 2014; Ribeiro et al. 2015). In addition, any extract contains high levels of various phytochemicals (phenols, flavonoids, alkaloids, and saponins). These phytochemical components are responsible for the extract’s biological activity (Sharifi-Rad et al. 2022b). Plants of the Zygophyllaceae family include the genus *Fagonia* about eighteen species are included (Täckholm 1974). It has been reported that this genus of plants was utilized as medicine by Bedouins and indigenous people in Africa. This flowering plant is typically found as perennial herbs or low shrubs; annuals are unusual (Boulos 2000). The genus *Fagonia* is found in desert wadis, calcareous coastal ridges, and sandy plains. It is found throughout Egypt’s Mediterranean coastal strip, the Sinai Peninsula, all of Egypt’s deserts, and the oases of the western desert. This species is widely used as a home remedy and its alcoholic and aqueous extracts are used as medications to treat ailments like kidney difficulties, diabetes, fever, asthma, toothaches, and stomach pain (El-Zayat 2002; Kamran et al. 2017). Numerous diseases, including hematological, neurological, endocrinological, and inflammatory disorders, have been treated using different portions of this herb. Additionally, a range of triterpenoids and saponins have been reported to be present, along with antioxidants. When used as a cooling agent, its infusion works well for stomatitis. It has a reputation for being a blood purifier. Additionally, the twigs of the plant are applied topically as a paste to neck swellings and tumors (Miyase et al. 1996; Khalik et al. 2000; Saeed and Sabir 2003). Other *Fagonia* species have been shown to have cytotoxic effect; for example, aqueous extracts of *F. indica* have been shown to be very effective against various types of cancer especially breast cancer (Khan et al. 2019). Lam et al. (2012) reported that aqueous extract of *F. indica* showed significant activity against MCF-7 breast cancer cells. High concentrations of phytochemicals may accumulate due to current environmental circumstances in plant tissues, which could have promising economic implications (Zahran and El-Amier 2014), and/or agricultural-industrial raw resources (Zahran and El-Amier 2013), and in the pharmaceutical sector (Zaki et al. 2016a, b). However, little is currently understood about little is currently understood about the comprehensive phytochemical profile and biological evaluation of *F. Arabica*, especially with regard to its aqueous extract. Finding a link between the identified polyphenolic compounds and their cytotoxic, antimicrobial, and antioxidant properties is the novel part of this study.

## Materials and methods

### Plant material

In April 2023, the aerial flowering parts of *Fagonia arabica* L. were collected from Wadi Hagul on Suez Road, Egypt (Supplementary Fig. 1). Its voucher herbarium specimen (CAIM-330) was deposited in the Herbarium of Flora and Phytotaxonomy Department, HRI, ARC, Agricultural Museum, Dokki, Giza, after the plant sample was verified by Prof. Dr. Abd El-Halim Abd El-Mogali Mohamed, chief researcher, Flora and Phytotaxonomy researches department, Horticulture Research Institute (HRI), Agricultural Research Center (ARC), Agricultural Museum, Dokki, Giza. The fresh aerial flowering parts of *F. arabica* were washed with refined water. Finally, the fresh parts were dried in the shade at room temperature until their weight remained constant. Then stored in dry glass containers at room temperature for later use.

### Experimental analytical procedures

#### Extraction of plant material

Air-dried aerial parts of *F. arabica* (300 g) were crushed into fine powder. They were sieved and mashed through mesh 60 before being kept for analysis. The crushed plant materials were soaked in 3000 mL (w/v) hot distilled water at 40 °C for three days, stirring occasionally. The residue was extracted once more using the same process after the extract was run through Whatman No. 1 filter paper and additional fresh hot distilled water until all the plant material had been removed. After collecting the filtrate, and reduced pressure. The thick semisolid gummy-type extract was freeze-dried and kept in a brown, airtight container at 4 °C until it was time for analysis (Aldalin et al. 2024).

### Qualitative phytochemical analysis

Aerial flowering parts of *F. arabica* was subjected to a preliminary phytochemical study using qualitative methods as outlined by (Trease and Evans 1989; Sofowara 1993; Haggag and Elhaw 2022; Abdelaziz et al. 2023; Mekky et al. 2024) to check for the presence of anthraquinone, glycosides, cardiac-glycosides, terpenoids, diterpenes, amino acids, proteins, alkaloids, flavonoids, steroids, saponins, tannins, phenols, and carbohydrates.

### Quantitative estimation of phytochemicals

#### Total phenolic acids

The total amount of phenolic acids in aerial flowering parts of *F. arabica* was determined using the Folin-Ciocalteu technique as described by (Chandra et al. 2014; Mahmoud et al. 2024), the total phenolics in aerial flowering parts of *F. arabica* was quantified. To prepare the plant aliquot and standard, the following were added: A solution was made by mixing 0.2 mL of Folin-Ciocalteu reagent (0.5 M) with 0.6 mL of purified water. After 15 min of vigorous shaking, an 8% w/v Na_2_CO_3_ solution (1.0 mL) was added to the mixture. Then further diluting to a volume of 3.0 mL, the mixture was left to incubate for half an hour in darkness. A wavelength of 725 nm was applied to measure the light absorption intensity of the resultant solution (PD-303UV, U.S.A), which had a deep blue color. The study was carried out three times. We used a method to calculate the total phenolics by the standard sample calibration curves equation Y = 0.003x + 0.0239, with an R^2^ value of 0.9942. The concentration ranges of gallic acid in methanol were 10, 20, 30, 40, and 50 µg mL^− 1^. Using the formula CV/m, where “C” refers to concentration in mg/mL, “V” for volume of the extract/mL, and “m” for the extract mass/g, the result was presented as gallic acid equivalent. GA eq/g. This data was derived from the standard graph equation.

#### Total tannins

The total tannins were assessed by the Folin-Denis spectrophotometer procedure created by Makkar (2013). In a cold environment, 0.5 mL of plant test and 0.5 mL of refined water were added to 0.1 g of polyvinyl poly pyrrolidone to accelerate the tannins. The tubes underwent a 4-hour incubation period at 4 °C before being centrifuged for 10 min. The only phenolics in the supernatant are those that don’t contain tannin. Take 100 µL of the phenolic non-tannin separate from the example and 500 µL of the Folin-Ciocalteu reagent (1 N). Fill every tube, including the clear one, to a capacity of 1000 µL with refined water. Once all the tubes are connected, let them sit for 5 min at ambient temperature. At that point, fill each test tube with 2.5 mL of 5% Na_2_CO_3_, including the clear. After thoroughly mixing the tubes once more, allow them to sit in total black for 40 min at ambient temperature. A spectrophotometer (PD-303UV, U.S.A) measuring at 725 nm was used to estimate the standard break up in methanol, and the adjustment bend of tannic acid was employed. The experiment was tripled. By utilizing the calibration graph equation (Y = 0.0086X + 0.0233, R2 = 0.9926) and the CV/m formula for phenol, the amount of tannin was calculated and reported in mg TAeq/g dry weight. The tannins content was quantified using the subsequent calculation:

$$ \begin{aligned} & {\text{Total~tannins~}}\left( {\text{g}} \right) = {\text{~}} \\ & \quad {\text{Total~phenolics~content~}}\left( {\text{g}} \right) - {\text{~nontannin~phenolics~content~}}\left( {\text{g}} \right) \\ \end{aligned} $$

#### Total flavonoids

Using aluminum chloride calorimetric method first described by Aryal et al. (2019) we measured the total flavonoid content in aerial parts of *F. arabica*. The amount of yellowish-orange coloration produced by the flavonoid-aluminum chloride complex reaction is a key variable in this process. Briefly, 2 mL of purified water, 150 µL of NaNO_2_ (5%), and extract were combined. The incubation period was 5 min. After waiting another 5 min, A solution was made by combining 150 µL of 10% AlCl_3_ with 1 mL of NaOH (4%). The next step was to vortex the mixture and then incubate it at 40 °C for 15 min. Using the same method, we also made a quercetin standard solution of variable concentrations. The study was replicated three times. We used the estimated equation to determine the flavonoid content, y = 0.0034_X_ + 0.0135, with an R^2^ value of 0.9971. It was then displayed as a quercetin equivalent per mg (QE eq/g) using the CV/m formula, just like the phenolics demonstrated earlier.

#### Total flavonols

The flavonol content was measured using AlCl_3_ colorimetric technique outlined by Miliauskas et al. (2004). Combine 2 mL of AlCl_3_ with 6 mL of CH_3_COONa. Next, for two hours, the sample was preserved in an incubator set at 20 °C. At 440 nm in wavelength, the samples were examined with a UV-Vis spectrophotometer. The total flavonols were determined as the mean ± SD (*n* = 3; all samples were examined in three replications) and reported as mg (mg rutin/g) of rutin equivalent/g.

#### Total alkaloids

The total alkaloid content of aerial flowering parts of *F. arabica* was quantitatively determined using the method outlined by Harborne (1973). The dried plant powder samples (5 g) were combined with A mixture of 70% ethanol to glacial acetic acid (4:1). Following a minimum of 4 h of standing, the mixture was filtered. We gradually added a strong NH_4_OH to precipitate the alkaloid found in the supernatant. After filtering through pre-weighed filter paper, the alkaloids that encouraged were dried at 70 °C in an oven until a consistent weight was reached. The plant sample alkaloid content was determined and presented as mg/100 g.

#### Total saponins

Weighed 5 g of the dry powder and combined it with 100 mL of ethanol (20%). The suspension was stirred and heated to approximately 55 °C in a water bath for 4 h. After filtration, the remaining substance was extracted again using 100 mL of ethanol (20%). The mixed extracts were condensed in a water container to a volume of around 40 mL. The concentration underwent a washing process using diethyl ether, followed by extraction with n-butyl alcohol. The n-butyl alcohol separate was then washed with a 5% watery NaCl solution. The excess arrangement was at first bubbled in a water bath and consequently dried in the oven until the weight remains constant was accomplished (Otang et al. 2012). The concentration of saponins in each 100 g of dry weight was calculated and reported in mg.

#### Total steroids

To hydrolyze the sample, weigh out 5 g and boil it in a 50 mL solution of HCl for 30 min. Pour the solution into a separator funnel after straining it through Whatman filter paper. Add the same volume of ethyl acetate, mix well, and allow it to separate into separate layers. Remove the ethyl acetate layer and discard the aqueous layer, keeping it for potential future use in research. Heat concentrated amyl alcohol to extract the steroids after the ethyl acetate layer has evaporated at 100 °C for 5 min. The mixture becomes hazy. After weighing it with Whatman filter paper and letting it cool in a desiccator, measure it once more (Herborne 1973). For each 100 g of dry weight, steroid concentration was calculated and reported in mg.

### HPLC analysis

The aqueous extract of *F. arabica* was subjected to an HPLC analysis as follows: Agilent Technologies Inc., Santa Clara, CA, USA, provided the Agilent 1260 series HPLC system for the analysis of the aqueous extract. A C18 column (100 mm × 4.6 mm i.d., 5 μm) was applied to pass the separation. The mobile phase was made up of (A) acetonitrile at 0.6 mL/min; (B) methanol; and (C) water with 0.2% H_3_PO_4_. The following method was used to determine the gradient elute: 0–11 min (96% of A, 2% of B); 11–13 min (50% of A, 25% of B); 13–17 min (40% of A, 30% of B); 17–20.5 min (50% of B, 50% of C); and 20.5–30 min (96% of A, 2% of B). 284 nm was used as the detecting wavelength (UV detector). The column was kept at a steady temperature of 30 °C, and the injection volume was 20 µL. Compounds were determined through a comparison of their retention durations to those of actual standards. The chemical quantities were assessed using calibration curves.

### Biological studies

#### Testing organisms

The antibacterial test’s human pathogen microbial strains are all sourced from Al-Azhar University, Regional Centre for Mycology and Biotechnology in Nasr City, Cairo, Egypt. These pathogenic organisms include gram -ve bacteria such as *Escherichia coli* (ATCC8739), *Klebsiella pneumoniae* (ATCC2146), and *Acinetobacter baumannii* (ATCC 19606); the VITEK2 system was used to identify gram + ve bacteria such as *Staphylococcus aureus* (ATCC25923), *Staphylococcus epidermidis* (ATCC-12228), and *Enterococcus faecalis* (ATCC-51299). The activity was measured in millimeters (mm) as a function of the inhibition zone.

### Agar well diffusion method

Many human pathogenic bacteria were employed as test specimens, as noted by Saied et al. (2023). In nutrient broth, subcultures of pure cultures of test samples of microorganisms were performed. The strains were uniformly spread on sterile petri plates coated with Mueller Hinton agar. Plates with a diameter of 6 mm were drilled with a circular borehole using a sterile borer. *F. arabica* aqueous extract (100 µL) was added to the well to assess the antibacterial activity. The zones of inhibition were then identified after the plates were incubated for an entire night at 37 °C. Sterilized purified water served as -ve control, and amoxicillin/clavulanic acid (AMC), a common antibiotic for bacteria, was used as + ve control at a dosage of 2000 µg mL^− 1^. Visual detection of microbial proliferation was made. Three runs of each test have been conducted.

### Minimum inhibitory concentrations (MICs)

The procedure outlined in the Clinical Lab Standards Institute (US) Method recommendations was used to prepare the MICs of the aqueous extract. The Minimum Bactericidal Concentration (MBC) test was conducted on Mueller-Hinton Agar (MHA) plates, whereas the MIC test was conducted using standard broth microdilution methods on round-bottom microtiter plates with 96 wells. The concentration of the microbial sample was adjusted at 1.5 × 106 CFU mL^− 1^. The MIC test was performed by inoculating 100 mL of MHB with 100 ill of aqueous extract solution stocks (800 µg mL^− 1^), diluted twice, and commencing from columns 4 to 12. Column 4 had the highest level of the components under test, whereas column 12 of the microtiter plate had the lowest. Column 2 contained only medium, while column 1 had both media and bacterial inoculums as positive control. 30 µL of the resazurin solution, the microtiter plate was incubated for 24 h at 37 °C after being filled into each well. No changes in hue were observed. Bacterial development was indicated by pink or colorless, whereas the absence of bacterial growth was represented by blue or purple. The least concentrations at which no color change occurred were identified as the MICs. MBCs were measured after the test dilutions in the well that the color has not changed were sub-cultured and incubated on agar plates for 1 day. The MBC values were determined because the bacterial growth was inhibited by the lowest concentration. µg/mL is used to present the results (Balouiri et al. 2016).

### Resazurin solution

Khalifa et al. (2013) reported that a resazurin solution was generated at 0.02% (wt/vol). After that, 10 mL of purified water added to 0.002 g of resazurin salt powder, and the combination was vortexed. A Millipore-membrane filter (0.2 m) was used to filter the mixture. For two weeks, store the resazurin solution at 4 °C.

### Minimum lethal concentrations (MLCs)

The micro-broth dilution assay, as defined by Elkhawas et al. (2022) was used to assess the MLC of aerial blooming portions of *F. arabica* aqueous extract against tested pathogens. Each culture was cultivated in conditions that used plant extract; the extract-free group in line one served as -ve control, while the bacterial-free group in line two served as + ve control. For the MLC analysis, a twofold dilution treatment with varying concentrations (200–1.56 mg mL^− 1^) was select. The MLC was then determined by distribute overnight cultures of each treatment concentration onto agar plates.

### Antioxidant property

Antioxidant activity estimation applying the radical scavenging procedure for DPPH. The ability of aqueous extract of *F. arabica* to scavenge free radicals was assessed using DPPH procedure in aqueous extract of *F. arabica*. Basically, a 0.1 mM DPPH solution was produced using ethanol. Subsequently, 1 mL of this solution was combined with 3 mL of aqueous extract in ethanol at various concentrations (1.95, 3.9, 7.8, 15.6, 31.25, 62.5, 125, 250, 500, and 1000 µg/mL). The mixture took 30 min to recover at chamber temperature after vigorous shaking. Then, at 517 nm was estimated utilizing a Milton Roy spectrophotometer that was noticeable in the UV. With ascorbic acid acting as the standard reference compound, three replicates of the test were conducted (Patel et al. 2010). Utilizing the example’s IC_50_ esteem, which can be found utilizing a log measurement restraint bend, the grouping of the example expected to inhibit half of the DPPH free radical was determined. Increased production of free radicals was indicated by lower absorbance in the reaction mixture. Using the following equation, the percentage of the DPPH scavenging effect evaluation is:

$$ \begin{aligned} & {\text{Percentage}}\;{\text{inhibition}}\left( \% \right){\text{or}}\;{\text{effect}}\;{\text{of}}\;{\text{DPPH}}\;{\text{scavenging}}\left( \% \right) \\ & \quad = \left[ {{\text{A}}_{0} - {\text{ A}}_{{\text{1}}} } \right) \div {\text{A}}_{0} ]{\text{ }} \times 100. \\ \end{aligned} $$

where: A_0_ —abs. of control; A_1_— abs. of the specimen or reference.

### Cytotoxicity and anti-tumor assay

A549 ATCC CCL-185 lung cancer cell lines and Vero ATCC CCL-81 kidney normal cells can be used in a variety of cell cultures. We utilized the 3-(4,5-dimethylthiazol-2-yl)-2,5-di-phenyltetrazolium bromide (MTT) test to evaluate the cytotoxic effects of the aqueous extract. To create a dense monolayer, cells were cultivated in microtiter plates for 24 h at 37 °C. A density of 1 × 104 cells per well was used for the inoculation process. The growth fluids were taken out of the microtiter plates once a confluent cell layer had formed, and the cells were repeatedly rinsed with a washing medium to eliminate any remaining monolayer. Next, a growth medium with (2%) fetal bovine serum (RPMI medium) was used to introduce the aqueous extract in two-fold serial dilutions. Each concentration was added in 100 µl to a different well for the experiment; three wells were used as controls and only got the maintenance medium. We examined each cell for indications of cytotoxicity, including rounding, granulation, shrinkage, or partial to total breakdown of the monolayer membrane, after the microtiter plate had been incubated at 37 ◦C. About 20 µL of an MTT solution (5 mg mL^− 1^ in PBS) supplied by Bio Basic Canada Inc., Markham, ON, Canada, was applied to each well. The plate was set on a shaker and shaken for 5 min at about 150 rpm to make sure the MTT and medium were thoroughly mixed. To enable full metabolism of the MTT, the plate was then incubated with CO_2_ (5%) at 37 °C for 4 h. The medium was then thrown away. If required, paper towels were used to wipe the plate of any debris. After then, 0.2 mL of DMSO was used to re-dissolve formazan, the MTT metabolite. The mixture was shaken for five minutes at 150 rpm to guarantee that the formazan was completely dissolved and mixed with the solvent. At 560 nm, the absorbance was measured after any disruptions were finally removed at 620 nm. The number of cells and the optical density need to be directly correlated (Alley et al. 1988; Van de Loosdrecht et al. 1994).

Morphological Cell Viability and Proliferation.

Following 48 h, MTT Cell Proliferation and Viability Assay Kit (TACS) was used to detect cell proliferation and vitality. Vero and Cancer cell line survival curves were plotted from surviving fraction and chemical concentration. The determined response parameter was the IC_50_ value, which inhibits cell viability by 50%, was determined and using an inverted microscope, the cellular morphology was examined by exposure to a variety of *F. arabica* aqueous extract concentrations or as compared with the control group, the morphology of Vero ATCC CCL-81 cercopithecus aethiops kidney normal cells and A549 ATCC CCL-185 lung cancer cell lines was Images were taken after being analyzed for alterations.

### Statistical analysis

Minitab^®^ version 19 and Microsoft Excel version 365 were used for statistical studies using ANOVA and *t-*test at the 0.05 level of probability (Snedecor and Cochran 1982). Parametrically distributed quantitative data was gathered. An acceptable margin of error of 5% and a 95% confidence interval were determined.

## Results

### Phytochemicals analysis of *F. arabica*

In the current investigation, a preliminary phytochemical analysis was conducted on the medicinal plant *F. arabica.* The results are displayed in Table 1, revealing the existence of tannins, steroids, terpenoids, diterpenes, glycosides, saponins, flavonoids, phenols, alkaloids, fixed oils, carbohydrates, proteins and amino acids. On the other hand, it showed the absence of both cardiac glycosides and anthraquinones based on preliminary phytochemical tests.

**Table 1:** The preliminary phytochemical screening of *F. arabica* aerial flowering parts

Phytoconstituents	Result
Flavonoids	+
Cardiac glycosides	-
Alkaloids	+
Steroids	+
Saponins	+
Phenols	+
Anthraquinones	-
Glycosides	+
Diterpenes	+
Tannins	+
Terpenoids	+
Fixed oils	+
Carbohydrates	+
Proteins and amino acids	+

+ (presence of phytoconstituents); - (absence of phytoconstituents)

The results in Supplementary Fig. 2 showed the phytochemicals of *F. arabica* aerial flowering parts where total flavonoids, flavonols, phenolic acids, and tannins were 263.43 ± 0.67 mg QE g^-1^, 114.53 ± 0.99 mg RT g^-1^, 180.75 ± 1.12 mg GA g^-1^ and 82.01 ± 0.62 mg TA g^-1^, respectively. While total alkaloids, saponins and steroids were 2.97 ± 0.10 mg/100 g, 4.86 ± 0.17 mg/100 g and 2.09 ± 0.2 mg/100 g, respectively.

### HPLC analysis of *F. arabica* aqueous extract

**Fig. 1:**
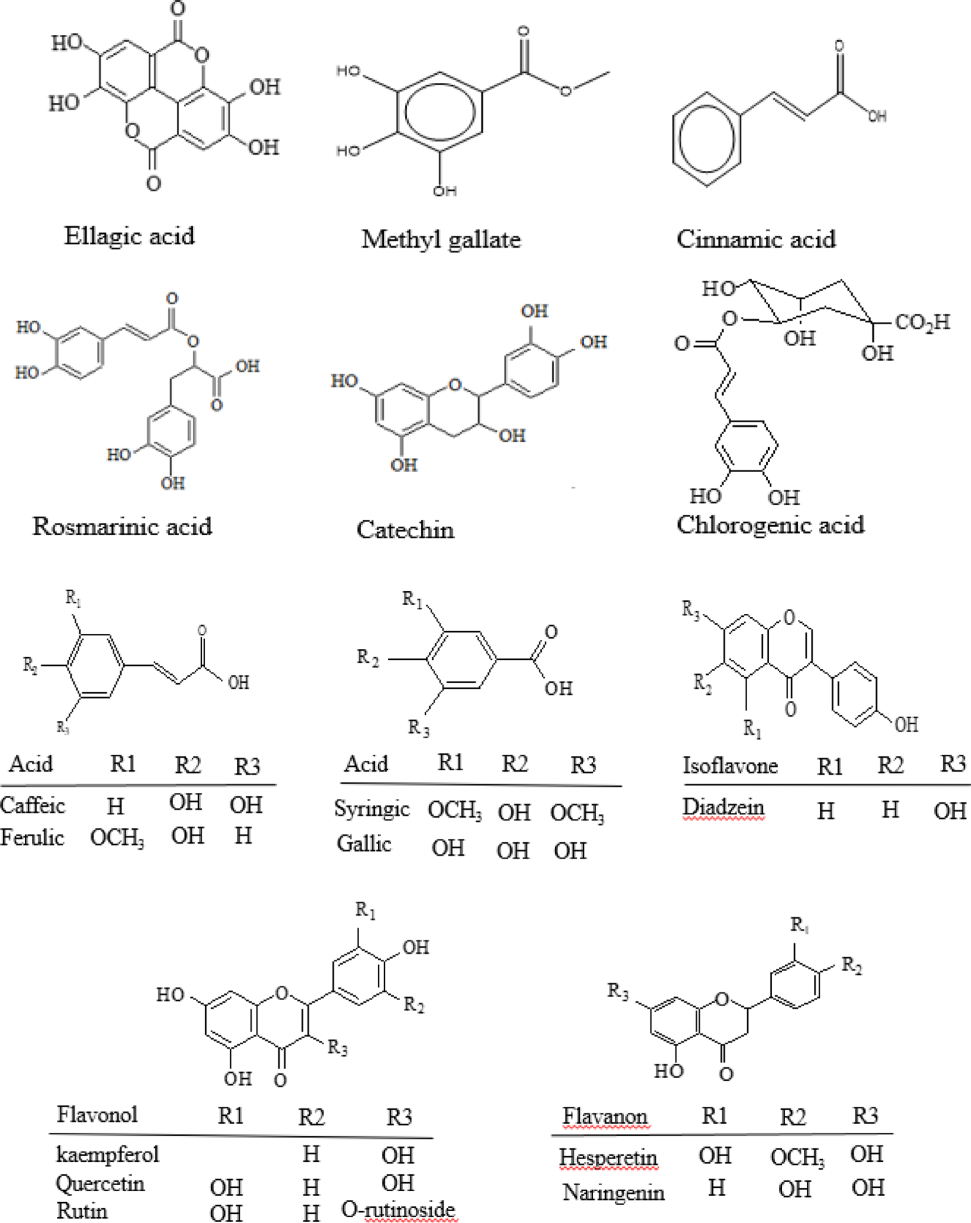
Phenolic compounds detected in the aqueous extract of the aerial flowering parts of *F. arabica* using HPLC

HPLC analysis of the aqueous extract of *F. arabica* aerial flowering parts revealed a variety of phenolic compounds. Supplementary Fig. 3, and Table 2 show the concentration and identification of fifteen phenolic compounds present in the aerial flowering parts of the aqueous extract of *F. arabica* namely: flavonoids such as rutin (12.09 µg mL^-1^), naringenin (13.91 µg mL^-1^), daidzein (6.12 µg mL^-1^), quercetin (7.1 µg mL^-1^), kaempferol (3.49 µg mL^-1^), hesperitin (4.6 µg mL^-1^), catechin (18.7 µg mL^-1^), phenolic acid and its derivatives such as gallic acid (19.39 µg mL^-1^), chlorogenic acid (21.6 µg mL^-1^), methyl gallate (9.67 µg mL^-1^), caffeic acid (16.9 µg mL^-1^), syringic acid (8.21 µg mL^-1^), ferulic acid (9.7 µg mL^-1^), rosmarinic acid (11.12 µg mL^-1^), and cinnamic acid (4.2 µg mL^-1^). Figure 1 shows the phenolic compounds detected in the aqueous extract of the aerial flowering parts of *F. arabica* using HPLC.

**Table 2:** Tentatively identified phenolic compounds from *F. arabica* aqueous extract using HPLC

Compounds	Concentration (µg/mL)	Retention time (Minutes)
Gallic acid	19.39	3.56
Chlorogenic acid	21.6	4.188
Catechin	18.7	4.599
Methyl gallate	9.67	5.618
Caffeic acid	16.9	5.99
Syringic acid	8.21	6.394
Rutin	12.09	6.925
Ferulic acid	9.7	9.739
Naringenin	13.91	10.164
Rosmarinic acid	11.12	11.506
Daidzein	6.12	15.995
Quercetin	7.1	17.379
Cinnamic acid	4.2	18.755
Kaempferol	3.49	20.014
Hesperetin	4.6	21.223

### Antioxidant activity of *F. arabica* aqueous extract

**Fig. 2:**
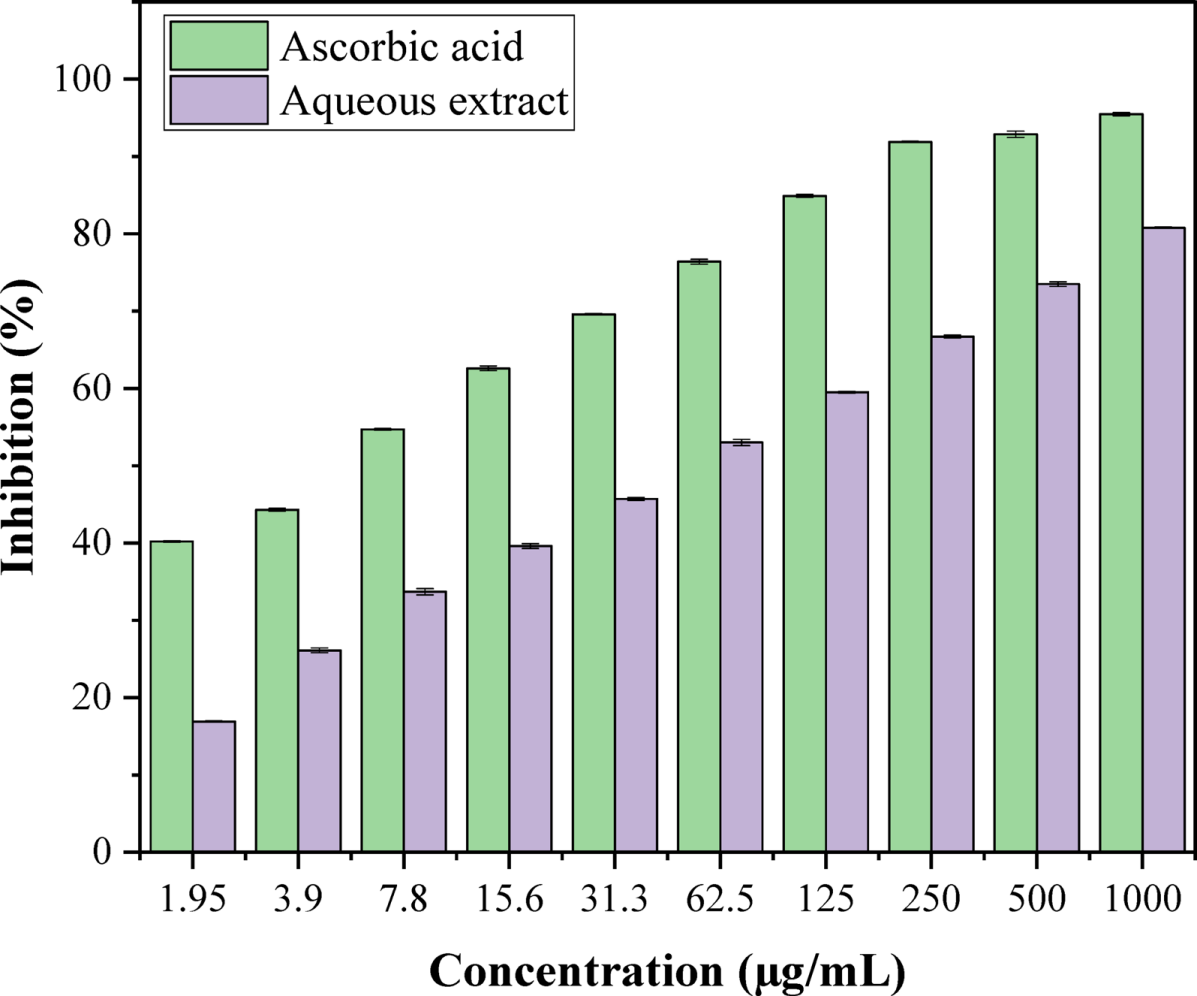
2,2 Diphenyl‑1‑picrylhydrazyl radical scavenging activity of ascorbic acid and aqueous extract of *F. arabica* aerial flowering parts. The average of the three replications + the standard error makes up the values

In the current study, the process of scavenging DPPH radicals of aqueous extract of *F. arabica* in comparison to known antioxidants (ascorbic acid) and half of the maximum amount that inhibits [IC_50_] of the radicals at various concentrations ranging from 1000 to 1.95 µg/mL are presented in Fig. 2. The DPPH scavenging percentage (antioxidant activity) of aqueous extract of *F. arabica* at concentrations of 62.5, 125, 250, 500, and 1000 µg/mL were 53, 59.5, 66.7,73.5 and 80.8%, respectively. The IC_50_ of ascorbic acid was significantly (*p* < 0.05) higher (4.81 µg/mL) comparison to aqueous extract was 46.25 µg/mL.

### Antimicrobial activity of *F. arabica* aqueous extract

**Fig. 3:**
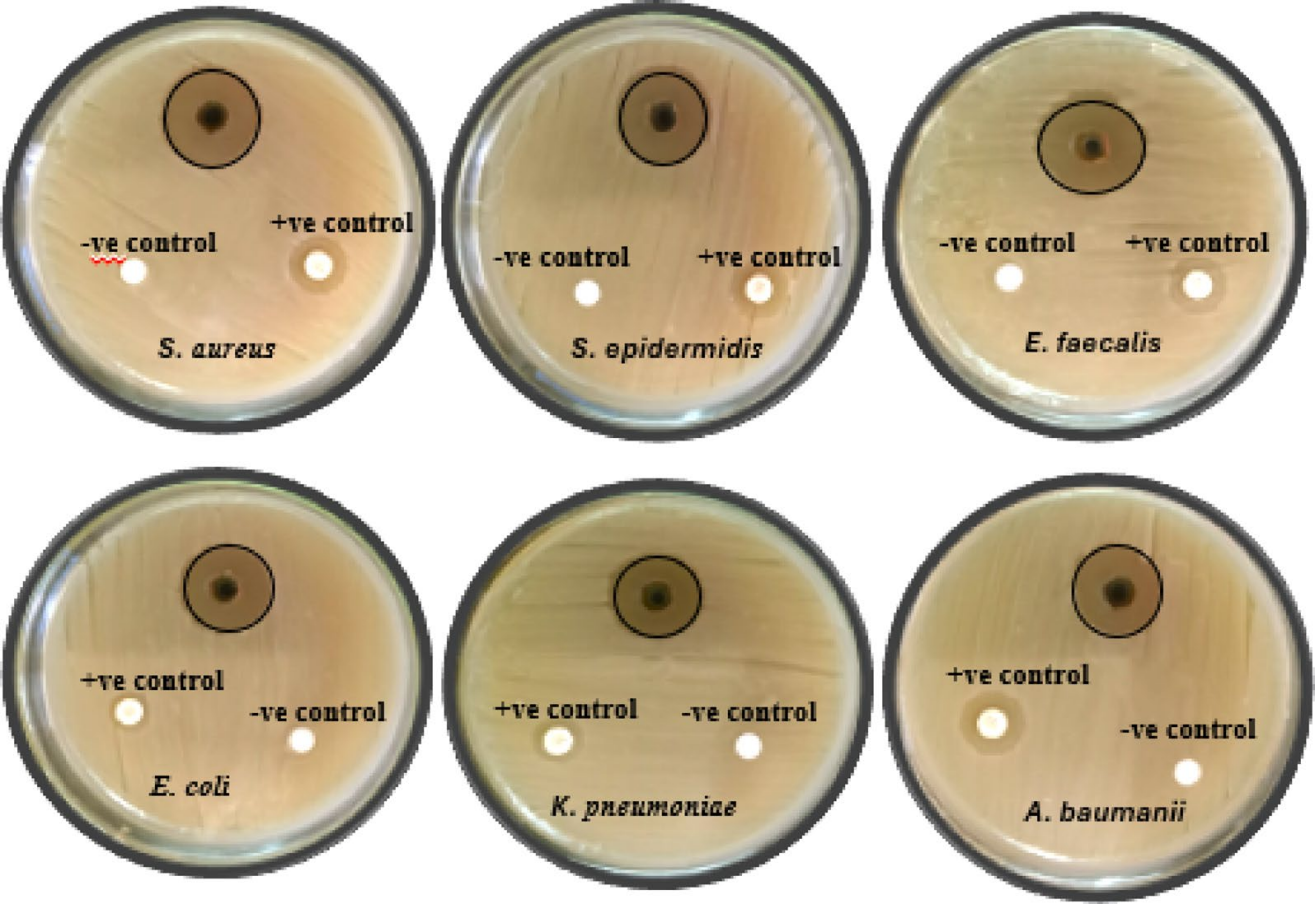
Inhibitory zones of *F. arabica* aqueous extract against pathogenic bacteria studied

The antimicrobial properties of six human pathogenic bacteria were evaluated in vitro using *F. arabica* aqueous extract. The inhibition zone results of this experiment indicated that the plant was effective against both Gram + ve and Gram -ve bacteria. Table 3; Fig. 3 showed that the aqueous extract of *F. arabica* at a concentration of 100 µg mL^− 1^ had antibacterial potential against all tested bacteria, with an inhibition zone ranging from 17 to 23 mm. The antibacterial potential was significantly (*p* < 0.05) increased with the addition of *F. arabica* extract. Gram -ve bacteria gave the lowest inhibition zone compared to Gram + ve bacteria. The MLCs and MICs of *F. arabica* against studied pathogenic bacteria are illustrated in Table 4; Fig. 3. MICs values of *F. arabica* ranged from 12.5 to 25 µg mL^− 1^ on *S. epidermidis*,* S. aureus*,* E. faecalis*,* E. coli*,* K. pneumoniae*, and *A. baumannii*, respectively. while the MICs ranged from 25 to 50 µg mL^− 1^ (Table 4 and Supplementary Fig. 7).

**Table 3:** Antibacterial activity of *F. arabica* aqueous extract against studied pathogenic bacteria

No.	Isolate Name	Diameter of Inhibition Zone (mm) by aqueous extract µL/mL		
		Aqueous extract	+ve control	-ve control
1	*Staphylococcus epidermidis* (ATCC-12228)	20 ± 0.75 ^a^	12 ± 0.47 ^ab^	0
2	*Staphylococcus aureus* (ATCC25923)	22 ± 0.47 ^a^	14 ± 0.38 ^ab^	0
3	*Enterococcus faecalis* (ATCC-51299)	23 ± 0.58 ^a^	15 ± 0.33 ^a^	0
4	*Escherichia coli* (ATCC8739)	21 ± 0.33 ^a^	11 ± 0.24 ^b^	0
5	*Klebsiella pneumoniae* (ATCC2146)	17 ± 0.62 ^a^	11 ± 0.33 ^b^	0
6	*Acinetobacter baumannii* (ATCC 19606)	18 ± 0.54 ^a^	12 ± 0.47 ^ab^	0

Data are presented as Mean ± SE for 3 replicates (*n* = 3). Indicating significantly different meaning values (*p* < 0.05)

**Table 4:** Minimum inhibitory concentration (MICs) and minimum lethal concentration (MLCs) determination of *F. arabica* aqueous extract against different human pathogenic bacteria

No.	Isolate Name	MICs and MLCs of aqueous extract by µg/mL			
		MICs		MLCs	
1	*Staphylococcus epidermidis* (ATCC-12228)		12.5 ± 0.53 ^b^	25 ± 0.54 ^b^	
2	*Staphylococcus aureus* (ATCC25923)		12.5 ± 0.31 ^b^	25 ± 0.33 ^b^	
3	*Enterococcus faecalis* (ATCC-51299)		12.5 ± 0.54 ^b^	25 ± 0.47 ^b^	
4	*Escherichia coli* (ATCC8739)		12.5 ± 0.31 ^b^	25 ± 0.44 ^b^	
5	*Klebsiella pneumoniae* (ATCC2146)		25 ± 0.75 ^a^	50 ± 0.75 ^a^	
6	*Acinetobacter baumannii* (ATCC 19606)		25 ± 0.58 ^a^	50 ± 0.58 ^a^	

Data are presented as Mean ± SE for 3 replicates (*n* = 3). Indicating significantly different meaning values (*p* < 0.05)

### Anti-cancer test of aqueous extract of *F. arabica*

**Fig. 4:**
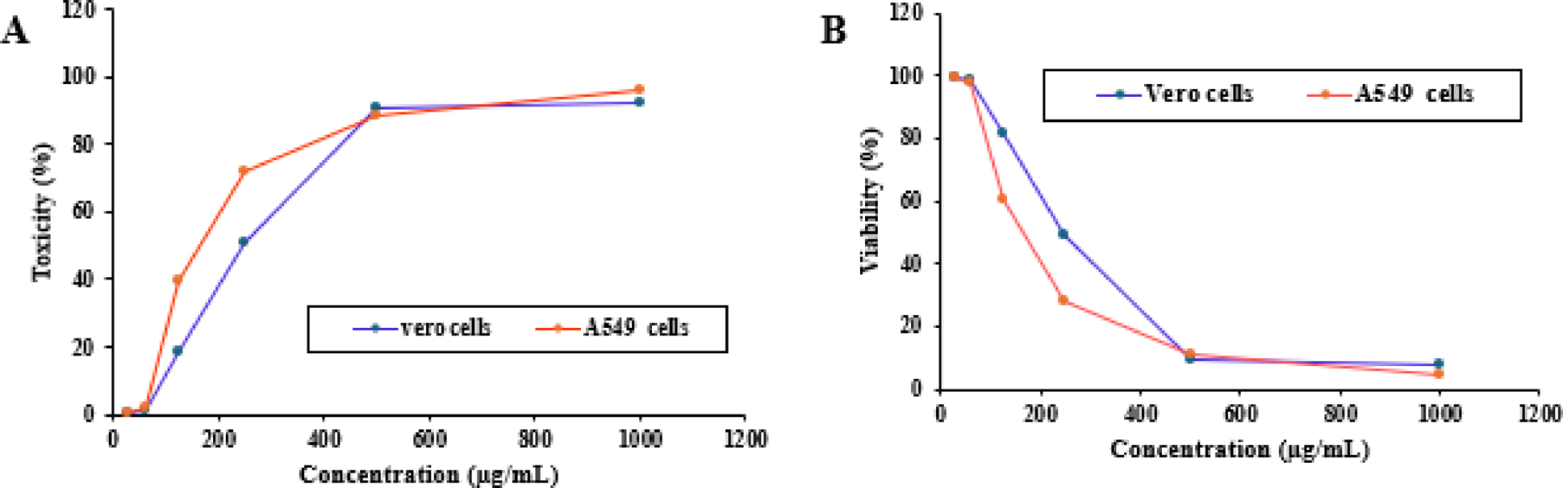
Effect of *F. arabica* aqueous extract onto Vero ATCC CCL-81 kidney normal cells and A549 ATCC CCL-185 lung cancer cell lines, (A) Toxicity; (B) Viability

**Fig. 5:**
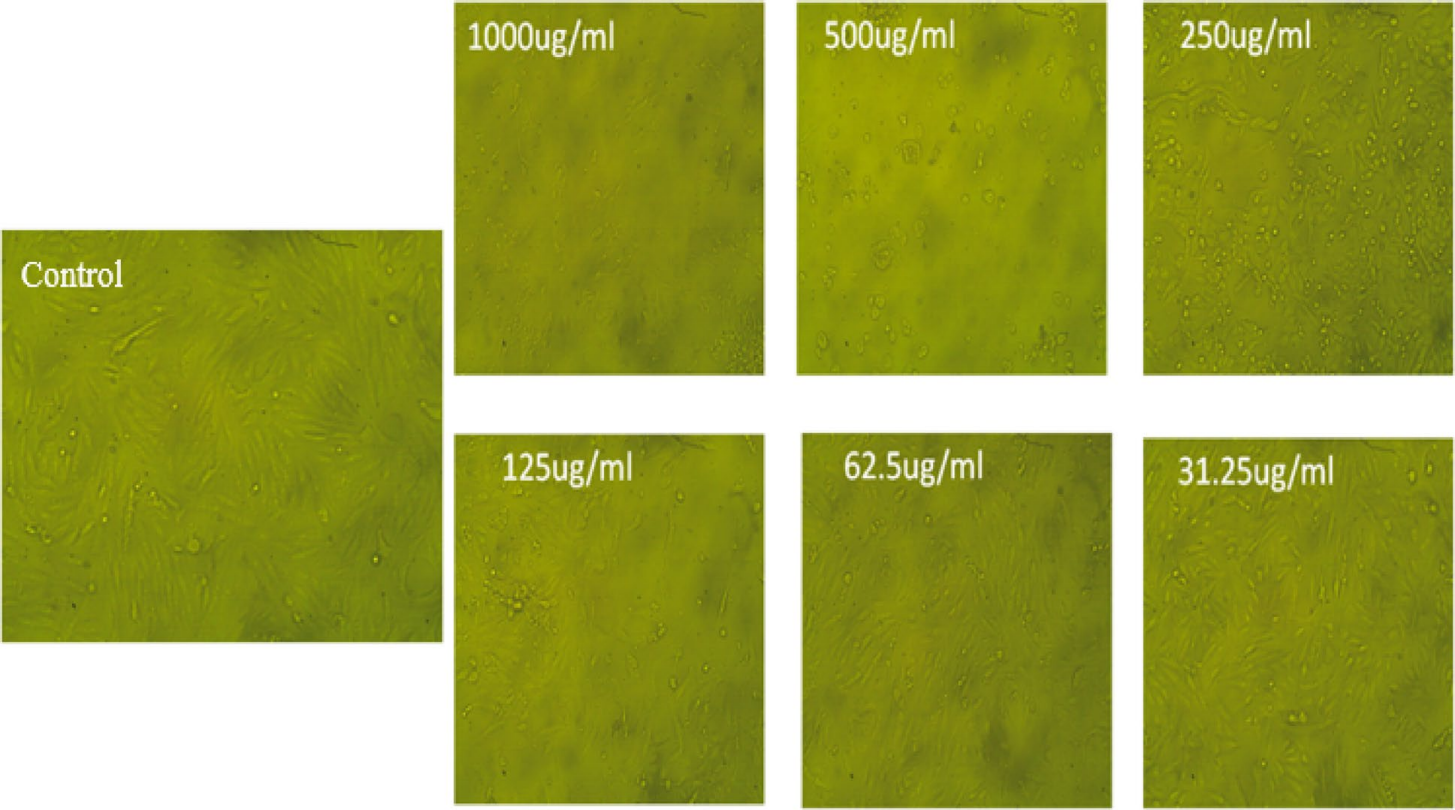
Morphological features of *F. arabica* aqueous extract on Vero ATCC CCL-81 kidney normal cells. The images taken from the cells were treated with an average size of 10 nm for 24 h

**Fig. 6:**
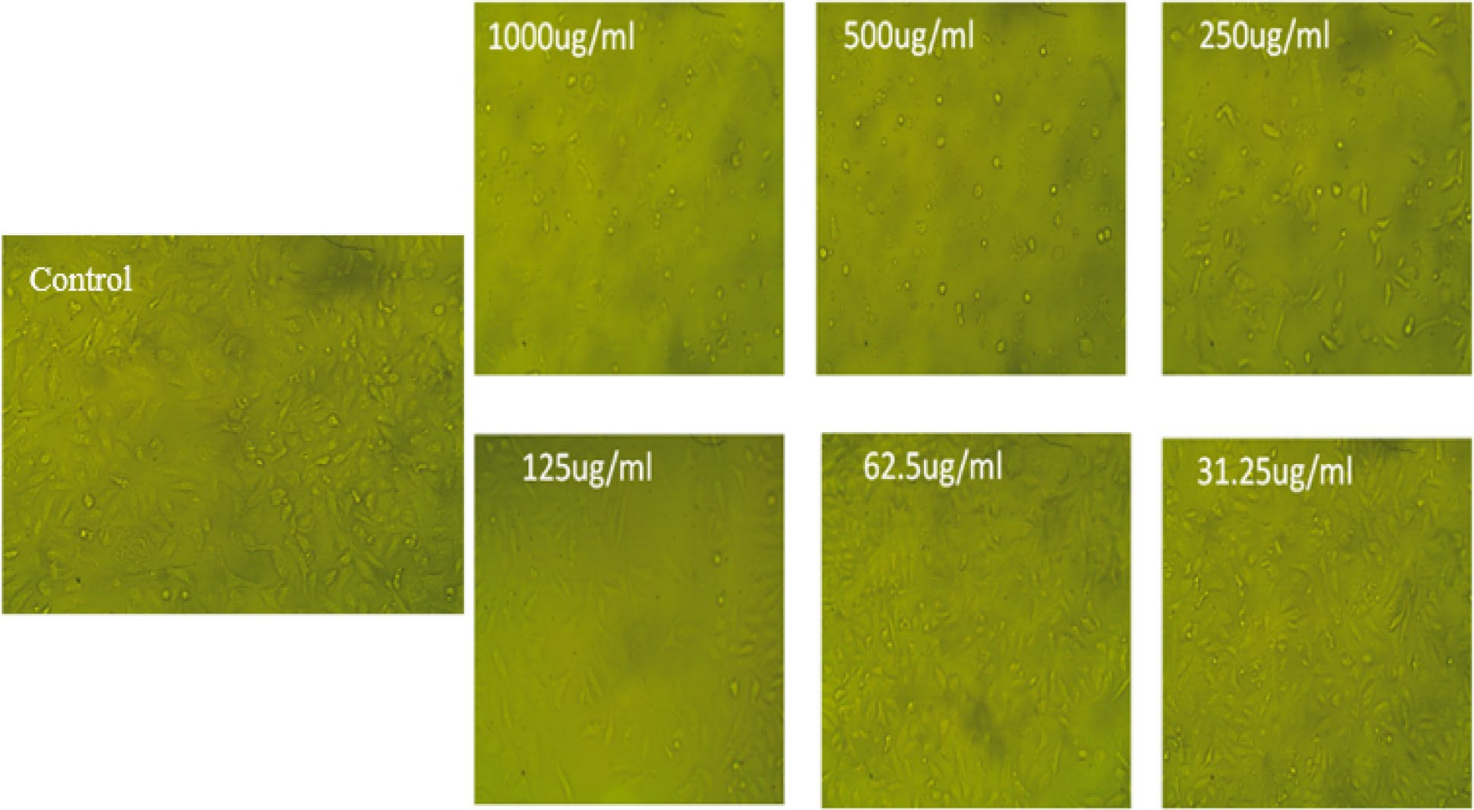
Morphological features of *F. arabica* aqueous extract on A549 ATCC CCL-185 lung cancer cell lines. The images taken from the cells were treated with an average size of 10 nm for 24 h

As shown in Fig. 4, the *F. arabica* aqueous extract showed low cytotoxicity against the Vero and A549 cell lines with IC_50_ values of 317.09 µg mL^− 1^ and 178.08 µg mL^− 1^, respectively. At doses of 250 µg mL^− 1^, the toxicity reached 51.03% for Vero cells and 71.84% for A549 cells. However, at doses of 1000 µg mL^− 1^, the toxicity reached 92.57% for Vero cells and 59.72% for A549 cells. As shown in Figs. 5 and 6, the microscope images of Vero and A549 cells treated with *F. arabica* aqueous extract for 24 h showed notable differences from control cells and morphological changes in cells such as cell shrinkage, apoptotic cells, nuclei condensation, and membrane blebbing.

## Discussion

Plant polyphenols are a class of compounds that exhibit strong antioxidant properties. They actively prevent dementia and cardiovascular disease and have antiviral and antitumor properties (Fraga et al. 2019). In the current research, a preliminary phytochemical screening for aerial flowering parts of *F. arabica* revealing the existence of various classes of secondary metabolites. The present results were in good agreement with the results of Fouda et al. (2022). The aerial flowering parts of *F. arabica* showed high levels of total flavonoids, flavonols, phenolic acids, tannins, alkaloids, saponins and steroids (Supplementary Fig. 2). Similar results have been reported previously by (Benmohamed et al. 2023; Aldalin et al. 2024; Elkady et al. 2024). Flavonoids, polyphenols, and phenolic compounds are examples of natural antioxidants found in herbs and spices that scavenge free radicals and lessen or completely eradicate the negative consequences of oxidative stress. The aerial flowering parts of *F. arabica* aqueous extract demonstrated considerable DPPH scavenging property of > 80% in the free radical scavenging property assay used in this investigation. Alike results have been reported previously by Mohamed et al. (2023). Also, similar results were published by Abd Elkarim et al. (2021), who claimed that the high flavonoid and tannin content in the aerial sections of *Synadenium grantii* was principally due to the substantial antioxidant activity of the isolated compounds, as shown by NMR analyses. The presence of various phenolic compounds produced during development and detected by HPLC analysis, including gallic acid, quercetin, caffeic acid, chlorogenic acid, and catechin, is linked to the antioxidant property of *F. arabica* extract in this study (Krishnamoorthy et al. 2023).

Assessing the antibacterial qualities of plants in vitro is the first step towards discovering novel biomolecules derived from plants and developing environmentally acceptable methods of treating infectious diseases in humans. In this experiment, the aqueous extract of *F. arabica* appeared as the greatest property against all tested bacteria, with an inhibition zone ranging from 18 to 23 mm. Lower MIC and MBC values ​​indicate higher antibacterial activity of the extracts on the tested bacterial strains (Sharifi-Rad et al. 2015, 2020). Similarly, Eman et al. (2010) found that *F. arabica* extract is effective against both Gram + ve and Gram-ve bacteria. The inclusion of *F. arabica* extract enhanced the antibacterial activity, presumably as a result of its high concentration of phenolic and bioactive components, including alkaloids, flavonoids, and saponins (Table 1 and Supplementary Fig. 2). The results were consistent with those of Pareek et al. (2012), who suggested that the plant extract’s diverse antibacterial activity is explained by the presence of numerous biologically active compounds, such as alkaloids, flavonoids, and saponins. Because the periplasmic zone and the layer of lipopolysaccharides surrounding Gram -ve bacteria shield their membrane from the harmful effects of plant extract, the results demonstrated that Gram -ve bacteria gave the lowest inhibition zone when compared to Gram + ve bacteria (Elsharkawy et al. 2019; Sharaf et al. 2022; Elkhateeb et al. 2024).

Abnormal morphological characteristics, including apoptotic cells, nuclei condensation, cell shrinkage, and membrane blistering, were discovered during the MTT experiment. These are all characteristics of cells undergoing apoptosis. The test samples may cause apoptosis in Vero and A549 cancer cell lines, according to MTT and microscopic analyses (Figs. 8, 9 and 10). Reactive oxygen species (ROS) have an impact on mitogenic signaling pathways and control cell growth. Accordingly, they cause unchecked cell division, which leads to the development of cancers and the start of carcinogenesis (Pandey et al. 2021). Accordingly, compounds with antioxidant qualities can lessen oxidative stress, which is why they are utilized to fight cancer (George and Abrahamse 2020). When the plant extract was administered to lung and kidney cells at concentrations of 178.08 ± 3.2 and 317.09 ± 5.57 µg/ml, respectively, it was found to be effective in reducing the viability of cancer cells by around 50%. IC_50_ value of *Fagonia* aqueous extract is greater than 90 µg/ml, it is generally considered low cytotoxic (Ioset el al. 2009). The high concentration of flavonoids and polyphenols in *F. arabica* may be the reason for its strong anticancer properties (Table 1 and Supplementary Fig. 2). This supports the findings of Aggarwal and Shishodia (2006), who discovered that polyphenolic compounds in plant extracts have an impact on genes necessary for cancer cell growth, death, and cell cycle regulation. Szliszka et al. (2008) discovered that flavonoids cause apoptosis in cancer cells by increasing their susceptibility to anticancer treatments in vitro, such as those involving members of the tumor necrosis factor (TNF) family, also referred to as death ligands. Strong antioxidant components and the capacity to destroy cancer cells are among the many biological qualities attributed to plants that contain flavonoids and polyphenols (Burits and Bucar 2000; Shylesh and Padikkala 2000; Alfawal et al. 2022). The anti-tumor effect of the aqueous extract may be due to its high flavonoid content. Flavonoids inhibit the growth of tumors and the proliferation of certain cancer cells in animal models. Certain flavonoids found in food have anticancer effects. The hydroxyl pattern of their B ring appears to have a major influence on the effects of flavones and flavonols, such as quercetin, especially inhibition of protein kinase activity and anti-proliferation (Kanadaswami et al. 2005). These promising results could be the beginning of further studies that could find plant *Fagonia arabica* to be a medicinal one.

## Conclusion

The findings demonstrated that a range of phenolic and flavonoid components were detected by HPLC analysis of the aqueous extract of the aerial flowering parts of *F. arabica*. The most significant and abundant of these compounds were catechin, gallic acid, chlorogenic acid, caffeic acid, and naringenin. With an inhibitory zone spanning 18 to 23 mm, the aqueous extract of *F. arabica* demonstrated the highest efficacy against all tested bacteria in this assay, including *Escherichia coli*,* Enterococcus faecalis*,* Acinetobacter baumannii*,* Staphylococcus aureus*, and *Staphylococcus epidermidis*. A high level of free radical scavenging property was demonstrated by the *F. arabica* extract (IC_50_ = 46.25 µg mL^-1^). With IC_50_ values of 178.08 ± 3.2 and 317.09 ± 5.57 µg mL^-1^, respectively, the results also demonstrated that the plant extract was efficient in reducing the viability of cancer cells by around 50% when applied to lung and kidney cells. The study’s findings raise the prospect of employing *F. arabica* as a therapeutic plant in a new, sustainable way.

## Electronic supplementary material

Below is the link to the electronic supplementary material.

Supplementary Material 1

## Data Availability

No datasets were generated or analysed during the current study.
