# Supplementary material for: Insight into the biological activities of Fagonia Arabica L. and its phytochemical constituents
Source: AMB Express. 2025 Aug 1;15:114. doi: 10.1186/s13568-025-01918-1 (PMC12316655; doi:10.1186/s13568-025-01918-1)
Supplement: Supplementary file 1 — Supplementary Material 1 [file 13568_2025_1918_MOESM1_ESM.docx]

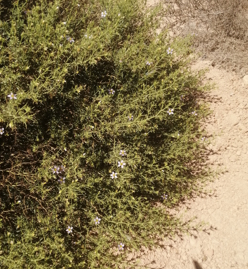


**Fig 1.** Aerial flowering parts of *Fagonia arabica* (L.) D C. (Dhamasa).


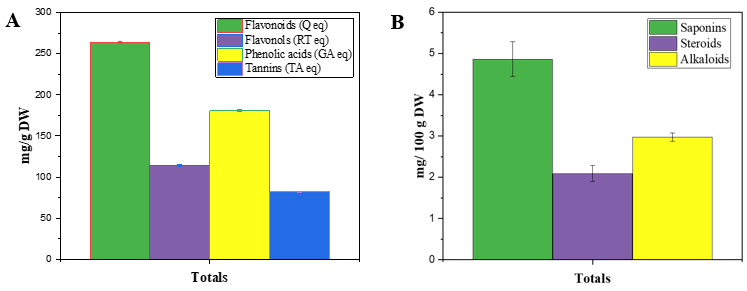


**Fig 2.** Quantitative analysis for phytochemicals of *F. arabica* aerial flowering parts; total flavonoids, flavonols, phenolic acids, tannins (A); total alkaloids, saponins, steroids (B).


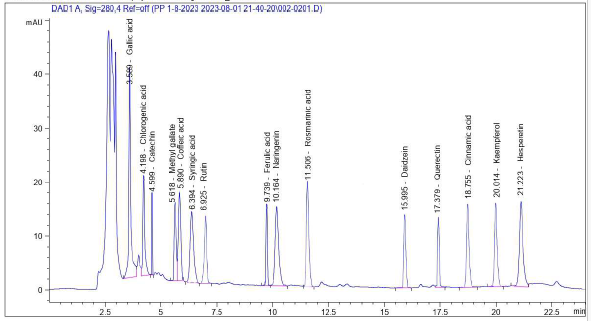


**Fig 3.** HPLC chromatogram shows the phenolic and flavonoid compounds that found in aerial flowering parts aqueous extract of *F. arabica*.


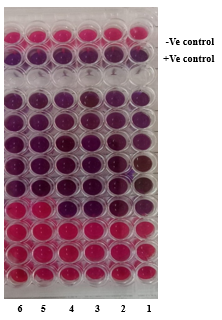


**Fig 7.** Minimum inhibitory concentrations and minimum lethal concentrations of *F. arabica* aqueous extract by Resazurin technique.
